# Estimating Causal Effects of Long-Term PM_2.5_ Exposure on Mortality in New Jersey

**DOI:** 10.1289/ehp.1409671

**Published:** 2016-04-15

**Authors:** Yan Wang, Itai Kloog, Brent A. Coull, Anna Kosheleva, Antonella Zanobetti, Joel D. Schwartz

**Affiliations:** 1 Department of Environmental Health, Harvard T.H. Chan School of Public Health, Boston, Massachusetts, USA; 2 Department of Geography, Ben Gurion University, Beer-Sheva, Israel; 3 Department of Biostatistics, Harvard T.H. Chan School of Public Health, Boston, Massachusetts, USA

## Abstract

**Background:**

Many studies have reported the associations between long-term exposure to PM2.5 and increased risk of death. However, to our knowledge, none has used a causal modeling approach or controlled for long-term temperature exposure, and few have used a general population sample.

**Objective:**

We estimated the causal effects of long-term PM2.5 exposure on mortality and tested the effect modifications by seasonal temperatures, census tract–level socioeconomic variables, and county-level health conditions.

**Methods:**

We applied a variant of the difference-in-differences approach, which serves to approximate random assignment of exposure across the population and hence estimate a causal effect. Specifically, we estimated the association between long-term exposure to PM2.5 and mortality while controlling for geographical differences using dummy variables for each census tract in New Jersey, a state-wide time trend using dummy variables for each year from 2004 to 2009, and mean summer and winter temperatures for each tract in each year. This approach assumed that no variable changing differentially over time across space other than seasonal temperatures confounded the association.

**Results:**

For each interquartile range (2 μg/m3) increase in annual PM2.5, there was a 3.0% [95% confidence interval (CI): 0.2, 5.9%] increase in all natural-cause mortality for the whole population, with similar results for people > 65 years old [3.5% (95% CI: 0.1, 6.9%)] and people ≤ 65 years old [3.1% (95% CI: –1.8, 8.2%)]. The mean summer temperature and the mean winter temperature in a census tract significantly modified the effects of long-term exposure to PM2.5 on mortality. We observed a higher percentage increase in mortality associated with PM2.5 in census tracts with more blacks, lower home value, or lower median income.

**Conclusions:**

Under the assumption of the difference-in-differences approach, we identified a causal effect of long-term PM2.5 exposure on mortality that was modified by seasonal temperatures and ecological socioeconomic status.

**Citation:**

Wang Y, Kloog I, Coull BA, Kosheleva A, Zanobetti A, Schwartz JD. 2016. Estimating causal effects of long-term PM2.5 exposure on mortality in New Jersey. Environ Health Perspect 124:1182–1188; http://dx.doi.org/10.1289/ehp.1409671

## Introduction

Many studies have reported the association of long-term exposure with fine particulate matter (PM_2.5_) with mortality by following cohorts of subjects over time (Beelen et al. 2008; Dockery et al. 1993; Jerrett et al. 2013; Krewski et al. 2009; Lepeule et al. 2012; Pope et al. 1995; Puett et al. 2009). Initial studies [the Harvard Six Cities (HSC) and the American Cancer Society (ACS) study] contrasted exposure across cities of residence (Dockery et al. 1993; Pope et al. 1995), and, more recently, land-use regression has been used to assign exposure, such as in the ACS Cancer Prevention II study (CPS-II) and the Nurses’ Health Study (NHS) (Jerrett et al. 2013; Puett et al. 2009).

However, a number of issues remain unresolved. First, the cohorts were convenience samples, which are not representative of the population as a whole and often underrepresent minorities. For example, both the ACS cohort and the NHS cohort examined populations with considerably higher levels of education than average (Pope et al. 1995; Puett et al. 2009). In addition, most cohorts (HSC, ACS, CPS-II, NHS) restricted the study population to city dwellers (Jerrett et al. 2013; Krewski et al. 2009; Lepeule et al. 2012; Puett et al. 2009), raising further issues about generalizability to the whole population. Second, temporal resolution of exposure has been limited. Because many land-use regression models rely on extensive monitoring in a single year (Henderson et al. 2007; Hoek et al. 2008) to supplement routine monitoring, they are only capable of estimating exposure for 1 year, which is taken as typical. Hence, only spatial variations in exposure can be used. In other studies, which used routine monitoring (Lepeule et al. 2012; Miller et al. 2007; Pope et al. 2009), lack of monitoring for PM_2.5_ likewise limited exposure contrasts to geographic variations because the PM_2.5_ level at the nearest monitoring site was assigned, and often, only a few monitoring sites were available for each city. This limitation makes control for geographic confounders critical in all of these studies.

Further, the causal modeling approach has not been used to estimate the effects of long-term exposure to PM_2.5_ on mortality. To estimate causal effects, we need a counterfactual framework. Causal modeling seeks to estimate the difference in value of the expected mortality in the population under the exposure they received versus what it would have been had they received an alternative exposure. Because that counterfactual cannot be observed, various methods seek legitimate surrogates for the unobserved potential outcome. Randomized trials are one approach but are not feasible for environmental exposures. Causal methods in observational epidemiology seek alternative ways to estimate a substitute for the counterfactual outcome (Baiocchi et al. 2014; Hernán et al. 2008; Rubin 1997). One approach uses formal modeling techniques, such as inverse probability weighting and propensity scores, to make the exposure independent of all measured predictors and relies on the untestable assumption of no unmeasured confounding (Cole and Hernán 2008; Stampf et al. 2010). Another approach relies on natural experiments or “random shocks,” which are used as instrumental variables. The variation in such an instrumental variable is a subset of the variation in exposure that is believed to be independent of measured and unmeasured confounders. However, the assumption that exposure variations caused by the instrumental variable are randomly assigned with respect to all measured or unmeasured confounders is untestable and often relies on external information for justification. When using natural experiments or random shocks, some studies made use of the temporal variation in exposure caused by the random shock. For example, Clancy et al. (2002) compared the mortality rates before (1984–1990) and after (1990–1996) the ban on coal sales in Dublin, Ireland (Clancy et al. 2002). The ban is an instrumental variable that was related to a substantial reduction in air pollution after its implementation. It is likely that the ban or a change in policy was independent of measured or unmeasured variables that confounded the association between air pollution and mortality. Other studies relied on the spatiotemporal variation in exposure caused by the instrumental variable, an example of which is the difference-in-differences approach. For example, Card and Krueger evaluated the difference in fast-food employment in New Jersey between February 1992 (2 months before an increase in the minimum wage) and November 1992 (5 months after the increase) and compared it with the difference in fast-food employment between February and November 1992 in Pennsylvania, a neighboring state that did not change its minimum wage (Card and Krueger 1994). The increase in the minimum wage was a random shock. In other words, the authors estimated the difference in the change (difference) in employment over time between the two states. Measured or unmeasured factors that might have confounded the association between the minimum wage and fast-food employment at each point in time (e.g., education) might have varied between the two states, but as long as any temporal variation in such factors was comparable between the states, they would not confound the difference in the change in employment over time between the states. Therefore, if the untestable assumption that the change in the minimum wage was the only factor influencing the difference in the rate of change in fast-food employment between New Jersey and Pennsylvania was true, the difference in differences was unconfounded.

In this paper, we describe a variant of the differences-in-differences approach to estimate the causal relationship between annual average PM_2.5_ and mortality in > 1,900 census tracts within New Jersey during 2004–2009.

## Methods

### Mortality Data

Death certificates in New Jersey from 2004 to 2009, including age, race, and the census tract of residence at the time of death for each individual, were obtained from the New Jersey Department of Health (NJDOH 2013). We only considered all natural-cause deaths. People who died of external causes including injuries and poisoning were excluded [i.e., *International Statistical Classification of Diseases, 10th Revision* (ICD-10) codes S00 through U99]. We regarded census tract as the unit of the analysis and aggregated annual natural-cause death in each of the census tracts.

### Exposure Assessment

The exposure assessment was based on a previously published hybrid model incorporating daily satellite remote sensing data at 1 km × 1 km spatial resolution (Kloog et al. 2014a). Briefly, we made use of a new algorithm developed by the National Aeronautics and Space Administration–Multi-Angle Implementation to Atmospheric Correction (NASA-MAIAC). The MAIAC algorithm provides aerosol optical depth (AOD) data that allow us to use high-resolution 1 km × 1 km (versus currently available 10 km) AOD data. PM_2.5_ was predicted using a mixed model with AOD and spatial and temporal predictors including meteorology, land use, and point emission. For the whole prediction area, the northeastern United States, the mean out-of-sample *R* ^2^ values obtained from 10-fold cross-validation and slope of predictions were 0.88 and 0.99, respectively, suggesting excellent prediction ability. The annual PM_2.5_ of a census tract in a given year was computed by averaging the predicted daily PM_2.5_ over all 1 km × 1 km grids within that census tract in that year.

### Temperature

The daily mean air temperature at each 1 km × 1 km grid in New Jersey was estimated using a similar mixed, spatiotemporal-resolved, and satellite-based model with Moderate Resolution Imaging Spectroradiometer (MODIS)-measured surface temperature in 1 km × 1 km spatial resolution (Kloog et al. 2014b). For the whole prediction area, the northeastern United States, the mean out-of-sample *R* ^2^ value obtained from 10-fold cross-validation was 0.95 when surface temperature was available and 0.94 when surface temperature was not, suggesting excellent prediction performance. Additional details have been published elsewhere (Kloog et al. 2014b). The mean summer temperature of a census tract in a given year was computed by averaging the daily predicted air temperature from June to August in that year over all 1 km× 1 km grids within that census tract, and the mean winter temperatures were the averages in January, February, and December. We controlled for the mean summer and winter temperatures when estimating the association between PM_2.5_ and mortality. These two variables were also tested as potential effect modifiers.

### Socioeconomic and Behavioral Data

From the U.S. Census for 2000, summary file 3, we obtained census tract–level data on population, socioeconomic status (SES) including percentage of black residents, median household income, and median value of owner-occupied homes (U.S. Census Bureau 2000). We also obtained age-adjusted yearly prevalence estimates of diabetes and smoking at the county level from 2004 to 2009 from the Centers for Disease Control and Prevention (CDC) Behavioral Risk Factor Surveillance System (BRFSS) (CDC 2013).

### Difference-in-Differences Approach

We begin with the potential outcomes framework of the Rubin Causal Model (Rubin 1991). Let *Y_c,t^A = a^_* be the potential outcome (aggregated number of deaths) in the population of census tract *c* if exposed to *A* = *a* in year *t*, and let *Y_c,t^A = a´^_* be the potential outcome under the alternative exposure *a´*. We would like to estimate *E*(*Y_c,t^A = a^_*)/*E*(*Y_c,t^A = a´^_*). We assume that the potential outcome depends on predictors in the following manner:

ln(*E*(*Y^a^_c,t_*)) = β_0_ + β_1_ *a* + β_2_ *Z_c_* + β_3_ *U_t_* + β_4_ *W_c,t_* + ln(*P_c_*), [1]

where *Z_c_* represents spatial confounders that vary among census tracts but not over the time period of the study (e.g., SES and diet), *U_t_* represents temporal confounders that vary over time but not among census tracts, *W_c,t_* represents confounders that vary over time and among census tracts, and ln(*P_c_*) is an offset term representing the natural log of the population in census tract *c*.

Although Equation 1 uses the aggregated number of deaths in a census tract in a year (in an ecological form), it is closely related to an individual-level model. Ecological bias is a potential concern when nonlinear dose–response relationships and within-area variability exist because an individual risk model may have a different form from the ecological model (Jackson et al. 2006). However, as shown by Lu and Zeger (2007), a model of aggregated event counts can be derived from an individual risk model when the exposure is common across individuals (Lu and Zeger 2007), as was the case for the present study, where PM_2.5_ for each individual during each year was assigned as the average value over all 1 km × 1 km geographic grids within their census tract in that year. Although such assignment introduces Berkson error in exposure assessment, it will not bias the effect estimates.

Specifically, for individual *i* in census tract *c* in year *t*, the risk of death (λ) could be modeled as follows:

λ*_ci_*(*t*, PM*_cit_*) = λ_0_ *_ci_*(*t*)exp(β_1_ PM*_cit_*) = λ_0_ *_ci_*exp(β_1_ PM*_cit_* + γ*_cit_*), [2]

where λ_0_ represents the baseline risk of mortality, and γ represents the individual-level confounders. Using the condition that PM*_cit_* = PM*_ct_*,

λ*_ci_*(*t*, PM*_cit_*) = λ_0_ *_ci_* exp(β_1_ PM*_ct_* + γ*_cit_*). [3]

This step introduces Berkson error. Then, we sum up both sides of Equation [3] over all of the subjects in tract *c* and year *t*,

μ*_ct_* = ∑*_i_*λ_0_ *_ci_* exp(β_1_PM*_ct_* + γ*_cit_*) = exp(β_1_PM*_ct_*) × ∑*_i_*λ_0_ *_ci_*exp(γ*_cit_*) = exp(β_1_PM*_ct_* + ln(∑*_i_*λ_0_ *_ci_*exp(γ*_cit_*))), [4]

where μ*_ct_* is the expected mortality in tract *c* in year *t*. Because ln(Σ*_i_*λ_0_ *_ci_*exp(γ*_cit_*)) is a function of *t* in tract *c*, we have

μ*_ct_* = exp(β_1_ PM*_ct_* + *f_c_*(*t*)), [5]

where *f_c_*(*t*) is a function of time for each census tract that could be decomposed into a tract-specific component that is constant over time (*Z_c_*), a time-varying component that is homogeneous over all tracts (*U_t_*), and a component that varies over time and among census tracts (*W_c_*_,_ *_t_*), which is essentially the same as Equation 1.

Then, let us look at Equation 1 again. If we look at differences between adjoining years, where the exposure in the other year is *a´*, we have the following:

ln(*E*(*Y_c,t_^^a^^*)) – ln(*E*(*Y^^a´^^_c,t_*_– 1_)) = β_1_(*a* – *a*´) + β_3_(*U_t_* – *U_t_*_– 1_) + β_4_(*W_c,t_* – *W_c,t_*_– 1_), [6]

and *Z_c_* and β_0_ have disappeared. If we then take the difference of these differences between census tracts *c* and *c´*, we have

[ln(*E*(*Y_c,t_^^a^^*)) – ln(*E*(*Y^^a´^^_c,t_*_– 1_))] – [ln(*E*(*Y_c´,t_^^b^^*)) – ln(*E*(*Y^^b´^^_c´,t_*_– 1_))] = β_1_[(*a* – *a*´) – (*b – b´*)] + β_4_[(*W_c,t_* – *W_c,t_*_– 1_) – (*W_c´,t_* – *W_c´,t_*_– 1_)], [7]

where *b* and *b´* are the exposures in tract *c´* at times *t* and *t* – 1, respectively. If the change in *W_c_*_,_ *_t_* over a year is the same in both locations, then (*W_c,t_* – *W_c,t_*_– 1_) – (*W_c´,t_* – *W_c´,t_*_– 1_) is zero, and the difference between locations in these within-location differences will only depend on the difference in their exposure differences; hence, this estimate will be causal. It is also a marginal, not a conditional, estimate because it is not conditioned on *Z_c_*, *U_t_*, and *W_c_*_,_ *_t_*. Alternatively, if differences in the rate of change of *W_c_*_,_ *_t_* are uncorrelated with differences in the rate of change of exposure in different locations, then the results are still causal. This is the key assumption of this approach. The advantage of this approach is that when this assumption holds, the ability to control for unmeasured confounders (*Z_c_*, *U_t_*, and *W_c_*_,_ *_t_*) need not be observed because they cancel out.

We can generalize this equation to include many census tracts instead of two, and to include 6 years instead of 2, and to deal with nonlinear changes over time. Estimating differences between years (Equation [6]) removes confounding by variables that vary by census tract but not by time (*Z_c_*). In the context of multiple tracts, we can accomplish this by controlling for indicator variables for each tract. Estimating differences between census tracts (Equation [7]) removes confounding by covariates that vary over time but are constant between census tracts (*U_t_*). Again, using indicator variables for each of the 6 years accomplishes the same thing even if the trend over time is not linear. More formally, from Equation 1, we have

ln(*E*(*Y_c,t_^^a^^*)) = β_0_ + β_1_ *a* + β_2_ *Z_c_* + β_3_ *U_t_* + β_4_ *W_c,t_* + ln*P_c_* = β_0_ + β_1_ *a* + Σ*__c__*__≠__ *__c__R__*β_2_ *Z_c_I_c_* + Σ*__t__*__≠__ *__t__R__*β_3_ *U_t_I_t_* + β_4_ *W_c,t_* + ln(*P_c_*) = β_0_ + β_1_ *a* + Σ*__c__*__≠__ *__c__R__*β*_c_I_c_* + Σ*__t__*__≠__ *__t__R__*β*_t_I_t_* + β_4_ *W_c,t_* + ln(*P_c_*), [8]

where *I_c_* and *I_t_* (indicator variables for tract *c* and year *t*, respectively) effectively control for *Z_c_* and *U_t_* under multi-tract and multi-year scenarios, in the same way that the differencing in Equations 6 and 7 controls for *Z_c_* and *U_t_* when there are only two tracts and two years. β*_c_* is the time-invariant component for tract *c*, and β*_t_* is time trend for year *t*. We used *c_R_* to denote the reference census tract and *t_R_* to denote the reference year. In summary, spatial and temporal confounders are controlled because differences among census tracts and time trends are controlled by *I_c_* and *I_t_*, and there is no confounding by person-specific factors that vary within years and census tracts because all persons in a census tract during a given year have the same exposure.

For the above to be a causal estimate, we must also assume that differences in *W_c_*_,_ *_t_* from the tract-level mean (captured by the dummy variable for tract) and the state-level trend are uncorrelated with the same differences in exposure. This is the untestable hypothesis, which must be judged on external information. How plausible is it? Factors such as SES and smoking rate vary across census tracts in New Jersey, and it is possible that these variations might be correlated with air pollution. But all differences between census tracts in any such variables are removed by using a dummy variable for each tract. What remains is variation in, for example, smoking rates that varied differentially among census tracts and over time. These variations would have to be correlated with variations in PM_2.5_ from the census tract average and mean yearly change in New Jersey for confounding to remain. This outcome seems highly implausible. Indeed, these tract-specific pollution changes mostly depend on EPA regulatory changes and on year-to-year variations in back trajectories (more- or less-polluted areas upwind), mixing height, and other meteorological factors that are unlikely to be related to smoking or to any other covariate over this 6-year time period, except temperature. Therefore, to account for potential confounding by temperature, we adjust for functions of temperatures as shown in Equation 9, where the difference-in-differences approach is modeled using Poisson regression with overdispersion (Donohue and Ho 2007):

ln(*E*(*Y_c,t_*)) = β_0_ + β_1_PM*_c,t_* + Σ*_c_*_≠_ *_c_R__*β*_c_I_c_* + Σ*_t_*_≠_ *_t_R__*β*_t_I_t_* + *s*(*Ts_c,t_*; **β*_Ts_***) + *s*(*Tw_c,t_*; **β*_Tw_***) + ln(*P_c_*), [9]

where PM*_c_*_,_ *_t_* is the PM_2.5_ concentration in tract *c* at time *t*, *I_c_* and *I_t_* represent indicators for each census tract and year, and *Ts_c_*_,_ *_t_* and *Tw_c_*_,_ *_t_* represent the mean summer and winter temperatures for each tract and year, which are modeled as linear splines (function *s*) with a single knot at their means to account for possible nonlinear associations of temperature with mortality. Seasonal temperatures are linked to mortality (Shi et al. 2015) and may also be related to aerosol concentration (Rosenfeld et al. 2014). Because an increase in temperature in the winter may have a different effect (and sign) on mortality than an increase in the summer, we chose to use the mean summer and mean winter temperature as two weather-related variables (as opposed to annual mean temperature) that may influence annual mortality rates (Shi et al. 2015). To summarize, the difference-in-differences approach controlled for *a*) geographical differences using dummy variables for each tract; *b*) a state-wide time trend using dummy variables for each year; and *c*) variables that varied differentially over time and across space that is correlated with PM_2.5_, which are seasonal temperatures. For the estimate to be causal, we assumed that no variable other than temperature that changed differentially across space and over time confounded the association between the exposure and the outcome.

The difference-in-differences approach was applied to estimate the causal effects of long-term exposure to PM_2.5_ on mortality among people in New Jersey. We also estimated the association for people > 65 years old and people ≤ 65 years old by stratification. We tested if the association was modified by the mean summer temperature and by the mean winter temperature. We performed this test by adding into the model two sets of product terms: one set comprised the product terms between the spline of the mean summer temperature and PM_2.5_, and the other set comprised the product terms between the spline of the mean winter temperature and PM_2.5_. We also tested if the association was modified by ecological SES variables at the census tract level using Census 2000 data (the percentage of black residents, the median household income, and median home values) and by ecological health condition at the county level using BRFSS data from 2004–2009 (age-adjusted prevalence of diabetes and smoking). These effect modifications were tested by adding a product term between PM_2.5_ and the modifier into the model. Not only did we test these effect modifications among the whole population, we also tested them in a subgroup analysis by restricting the study population to the white residents (70% of the total population) to determine whether the results were consistent within a race group. Consistency could reflect whether the association estimated using the whole population was confounded by individual-level race group. We did not repeat the analysis for other race groups owing to insufficient power to detect effect modifications. In addition, because these effect modifiers all reflected the SES of a census tract and were potentially related to each other, we fitted a model with simultaneous interactions of PM_2.5_ with percent of black residents, home value, household income, smoking rate, and diabetes rate to determine the most robust modifiers. We used backward elimination to select the modifiers. Specifically, we started with a model with all five interaction terms. Then, the interaction term with the largest *p*-value was dropped, and a model without that interaction term was refitted. We repeated this procedure and stopped dropping variables until each of the remaining interaction terms had a *p*-value < 0.05.

To compare the difference-in-differences approach with an estimate derived using only the within-tract variation of the exposure, we performed a sensitivity analysis fitting Poisson regression within each of the census tracts that regressed total mortality against PM_2.5_ and pooled the effect estimates using random-effects meta-analysis.

All statistical analyses were performed using R 3.1.2 (R Core Team 2014). Statistical significance was defined as *p*-value < 0.05.

## Results

Using population counts from Census 2000 data, we studied 1,938 census tracts in New Jersey during 2004–2009. In total, there were 365,530 deaths from 2004 to 2009, among which 281,170 deaths were at ages > 65, representing 77% of the total. Table 1 and Table 2 summarize the spatial and temporal variation of mortality, PM_2.5_, and temperature. The spatial variation of mortality was calculated by first averaging the annual deaths from 2004 to 2009 in each of the census tracts and then summarizing the distribution using these death counts. The spatial distribution of mortality had a mean of 31.4 deaths per year per census tract. Much of the variation in deaths was due to variations in the age distribution and size of the population in each tract. For example, the 5th–95th percentile range in the annual mortality rate of persons > 65 years old across census tracts was from 22.1 to 62.8 per thousand. The 5th–95th percentile range of average annual PM_2.5_ over 6 years ranged from 9.9 to 12.9 μg/m^3^ across census tracts with a mean of 11.3 μg/m^3^. The 5th–95th percentile range of mean temperature varied from 17.2°C to 19.6°C in summer, and from 4.6°C to 7.0°C in winter. The temporal trend is presented using the average of the variables over all of the census tracts in New Jersey in each year from 2004 to 2009. Mortality counts went down in 2006 and 2007 compared with 2004 and 2005, but they went back up slightly in 2008 and 2009, indicative of nonlinear or random pattern in temporal variation.

**Table 1:** Distribution of census tract–specific mean values for 2004 through 2009 for annual all natural-cause mortality, annual mean PM_2.5_, mean summer temperature, and mean winter temperature among 1,938 census tracts in New Jersey.

Variable	Mean	5th percentile	25th percentile	Median	75th percentile	95th percentile
Death counts per census tract per year (all age groups)	31.4	7.7	17.8	27.0	39.8	70.0
Mortality rate (all age groups, per 1,000)	7.3	3.0	4.9	6.6	8.5	13.6
Population [all age groups, based on Census 2000 data (U.S. Census Bureau 2000)]	4,412	1,853	3,152	4,181	5,562	7,527
Death counts per census tract per year (age > 65)	24.2	4.3	12.5	19.5	30.3	58.7
Mortality rate (age > 65, per 1,000)	40.1	22.1	31.2	38.5	47.2	62.8
Population [age > 65, based on Census 2000 data (U.S. Census Bureau 2000)]	598	175	350	525	756	1,207
Death counts per census tract per year (age ≤ 65)	7.2	2.0	4.5	6.7	9.3	14.8
Mortality rate (age ≤ 65, per 1,000)	2.1	0.8	1.3	1.8	2.4	4.2
Population [age ≤ 65, based on Census 2000 data (U.S. Census Bureau 2000)]	3,814	1,535	2,712	3,639	4,868	6,555
Annual PM_2.5_ (μg/m^3^)	11.3	9.9	10.8	11.2	11.9	12.9
Summer temperature^*a*^ (°C)	18.6	17.2	18.2	18.7	19.1	19.6
Winter temperature^*a*^ (°C)	5.9	4.6	5.6	5.9	6.2	7.0
^***a***^Summer (winter) temperature is an average of the predicted daily temperatures across all 1 km × 1 km grids in a given census tract during June, July, and August (January, February, and December) in a given year.						

**Table 2:** Annual mean values (± SD) across 1,938 New Jersey census tracts for all natural-cause mortality, annual mean PM_2.5_, mean summer temperature, and mean winter temperature.

Variable	2004	2005	2006	2007	2008	2009
Death counts per census tract per year (all age groups)	34.3 ± 23.9	34.2 ± 23.7	29.2 ± 22.5	28.7 ± 21.6	30.2 ± 21.3	32.0 ± 22.6
Death counts per census tract per year (age > 65)	26.4 ± 21.2	26.5 ± 21.0	22.2 ± 19.8	22.2 ± 19.1	23.2 ± 19.1	24.6 ± 20.2
Death counts per census tract per year (age ≤ 65)	7.9 ± 5.4	7.7 ± 5.2	7.0 ± 5.1	6.6 ± 4.7	7.0 ± 4.6	7.4 ± 4.7
Annual PM_2.5_ (μg/m^3^)	12.3 ± 1.0	12.8 ± 1.2	11.7 ± 0.9	11.6 ± 1.0	10.6 ± 0.8	9.1 ± 0.7
Summer temperature^*a*^ (°C)	18.1 ± 0.6	20.3 ± 0.8	19.1 ± 0.7	18.4 ± 0.7	18.6 ± 0.8	17.3 ± 0.7
Winter temperature^*a*^ (°C)	4.3 ± 0.7	5.0 ± 0.7	7.8 ± 0.6	5.9 ± 0.7	6.7 ± 0.7	5.7 ± 0.8
^***a***^Summer (winter) temperature is an average of the predicted daily temperatures across all 1 km × 1 km grids in a given census tract during June, July, and August (January, February, and December) in a given year.						

On the basis of the difference-in-differences approach (Equation 9), we found a 3.0% [95% confidence interval (CI): 0.2, 5.9%] increase in all natural-cause mortality for each interquartile range (IQR) increase in PM_2.5_ (2 μg/m^3^) among all residents in 1,938 census tracts in New Jersey during 2004–2009. By comparison, the meta-analysis pooling all within-census-tract effects showed a similar increase of 3.7% (95% CI: 2.9, 4.5%) in mortality per IQR increase in PM_2.5_. Restricting the study population to age of death > 65 years, we obtained a similar effect estimate: there was a 3.5% (95% CI: 0.1, 6.9%) increase in mortality per IQR increase in PM_2.5_. For people ≤ 65 years old, the percent change in mortality was similar, 3.1% (95% CI: –1.8, 8.2%), albeit with a wider confidence interval.

The percent change in mortality with an IQR increase in PM_2.5_ was 1.8% (95% CI: –1.6, 5.2%) if the mean summer and winter temperatures were at the average across all tracts and years (Table 3). By comparison, the percent change in mortality with an IQR increase in PM_2.5_ was –1.6% (95% CI: –4.2, 1.1%) if the mean summer temperature was 1°C below the average across tracts and years and the mean winter temperature was at the average (interaction *p*-value < 0.01); the percent change was 1.6% (95% CI: –0.6, 3.8%) if the mean summer temperature was 1°C above the average across tracts and years and the mean winter temperature was at the average (interaction *p*-value 0.73). The percent change in mortality was 1.6% (95% CI: –0.6, 3.9%) if the mean winter temperature was 1°C below the average across tracts and years and the mean summer temperature was at the average (interaction *p*-value 0.82); the percent change was 5.3% (95% CI: 2.9, 7.8%) if the mean winter temperature was 1°C above the average across tracts and years and the mean summer temperature was at the average (interaction *p*-value < 0.01).

**Table 3:** Percent change (95% confidence interval) in mortality per interquartile range increase (2 μg/m^3^) increase in PM_2.5_ at given summer and winter temperatures.

Mean summer temperature (˚C)	Mean winter temperature (˚C)	Percent change (95% CI) in mortality per IQR increase in PM_2.5_
18.6^*a*^ (Average)	5.9^*b*^ (Average)	1.8% (–1.6, 5.2%)
17.6 (Average – 1)	5.9 (Average)	–1.6% (–4.2, 1.1%)
19.6 (Average + 1)	5.9 (Average)	1.6% (–0.6, 3.8%)
18.6 (Average)	4.9 (Average – 1)	1.6% (–0.6, 3.9%)
18.6 (Average)	6.9 (Average + 1)	5.3% (2.9, 7.8%)
Abbreviations: CI, confidence interval; IQR, interquartile range ^***a***^Average of the census tract–specific mean summer temperature across 1,938 census tracts during 2004–2009. ^***b***^Average of the census tract–specific mean winter temperature across 1,938 census tracts during 2004–2009.		

**Figure 1:**
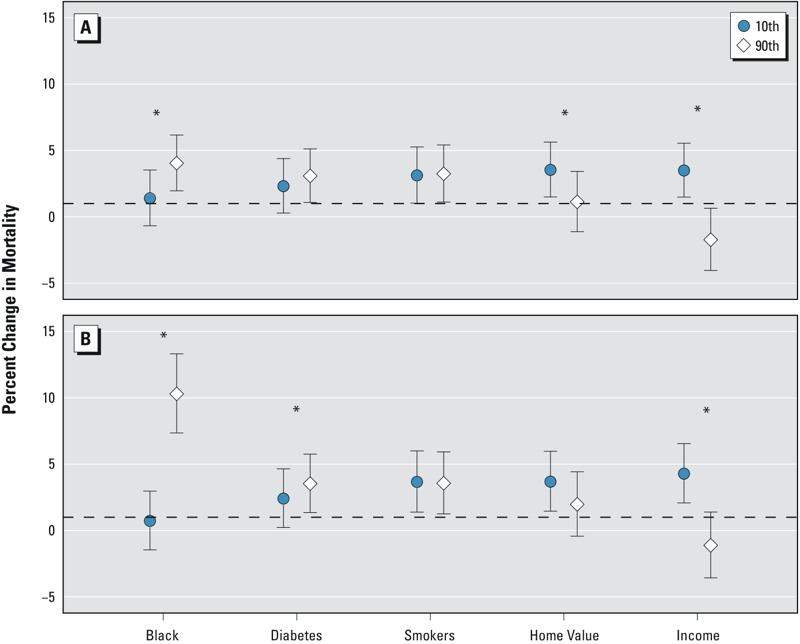
Percent change in mortality with 95% confidence intervals for each interquartile range (2.0 μg/m^3^) increase in PM_2.5_ at the upper and lower decile of each modifier: percent of black residents (10th percentile = 0.2%, 90th percentile = 52.0%), percent of persons with diabetes (10th percentile = 6.1%, 90th percentile = 9.2%), smoking rate (10th percentile = 7.8%, 90th percentile = 15.9%), median home value (10th percentile = 189,300, 90th percentile = 578,600 USD), and median household income (10th percentile = 35,625, 90th percentile = 115,049 USD) among (*A*) the whole population and (*B*) the white residents in New Jersey. Census tract–specific percent of black residents, median home value, and median household income came from Census 2000 data (U.S. Census Bureau 2000). County-level percent diabetics and smoking rate came from BRFSS data from 2004 to 2009 (CDC 2013). *Indicates interaction *p* < 0.05.

Figure 1 shows the estimated effects per IQR increase in PM_2.5_ on mortality rates in the upper and lower deciles of census tract–level percent of black residents, median home value, and median household income from Census 2000 data and age-adjusted diabetes and smoking rates from BRFSS data during 2004–2009. Among the whole population, the percent change in mortality associated with PM_2.5_ was modified by the percent of black residents (interaction *p* < 0.01), median income (interaction *p* < 0.01), and home values (interaction *p* = 0.02). We did not find effect modifications by smoking rate (interaction *p* = 0.60) or percent of persons with diabetes (interaction *p* = 0.06). Using backward elimination to select interaction terms from the simultaneous interaction model, we found that median household income was the only robust modifier that finally remained in the model. We also tested the consistency of these results among white residents (70% of the total population). We found that PM_2.5_ significantly interacted with percent of black residents (interaction *p* < 0.01), age-adjusted diabetes (interaction *p* < 0.01), and median income (interaction *p* < 0.01), but not with smoking rate (interaction *p* = 0.63) or median home value (interaction *p* = 0.13).

## Discussion

The present study used a variant of the difference-in-differences approach to estimate the causal effect of long-term exposure to PM_2.5_ on mortality in a large and general population.

We estimated the association between PM_2.5_ and mortality using a counterfactual framework. We accounted for SES, behavioral, and other risk factors that vary among census tracts by modeling dummy variables for each tract. We limited potential changes over time in such risk factors by focusing on a short time period (6 years) and by adjusting for average changes from year to year in New Jersey as a whole. If our assumption that yearly deviations from the state-wide yearly fluctuations in PM_2.5_ by tract (mostly resulting from regulatory and meteorological fluctuations) are unlikely to be associated with changes in other risk factors holds, we have identified a causal association.

The results add to the still relatively small body of literature that uses the general population, including both high and low SES individuals, all occupations, and both rural and urban residents.

We have identified interactions between PM_2.5_ and seasonal temperature. Very few studies have looked at the health effects of long-term temperature. An increase in the mean summer temperature, a decrease in the mean winter temperature, or an increase in the variability of summer or winter temperature was associated with a decrease in the risk of death among Medicare beneficiaries in New England during 2000–2008 (Shi et al. 2015). There are also very few studies that have investigated the interaction between long-term temperature and long-term PM_2.5_. A survival analysis among > 35 million Medicare beneficiaries residing in 207 U.S. cities during 2000–2010 found that an increase in annual, summer, or winter temperature was associated with an increase in the hazard ratio of death associated with PM_2.5_ (Kioumourtzoglou et al. 2016). We consistently found that an increase in the mean winter temperature was associated with an increase in the effects of PM_2.5_ on mortality. With regard to summer, the association between an IQR increase in PM_2.5_ and mortality in tracts with mean summer temperatures that were higher than the average was similar to the overall association. Here, the interaction was driven by a reduced risk of mortality in association with PM_2.5_ when mean summer temperatures were lower than the average. Under changing climate conditions, a rise in temperature not only would increase mortality through the direct effects of temperature but also would increase the effects of long-term PM_2.5_ exposure on mortality.

By analyzing the population of an entire state, we had sufficient power to test interaction and found that the effects of PM_2.5_ were greater in census tracts with a higher percentage of black residents, lower median home value, or lower median home income. Median household income was the most robust variable among these three SES variables. All of these analyses consistently suggested that the effects of PM_2.5_ were greater in tracts with lower SES. Consistent with our findings, in a recent study, Kioumourtzoglou et al. (2016) also found that a unit increase in PM_2.5_ in cities with higher percentages of black residents or lower household incomes was associated with a larger percent increase in mortality among > 35 million Medicare beneficiaries residing in 207 U.S. cities during 2000–2010 (Kioumourtzoglou et al. 2016). When restricting the analysis to white residents, we found that the interactions were basically consistent with the analyses for the whole population. This finding suggests that the estimates obtained using the whole population for PM_2.5_ were not confounded by individual-level race. The consistency between these two analyses also suggested that the SES of the neighborhood (or other people) would be associated with an individual’s susceptibility, which is a contextual effect.

We identified this association in a location and during a time period with low concentrations of PM_2.5_. The average PM_2.5_ over the period of study was 11.3 μg/m^3^, and the range across the census tracts was from 8.2 μg/m^3^ to 13.7 μg/m^3^. Hence, this association was estimated at PM_2.5_ levels completely below the old EPA annual standard of 15 μg/m^3^ (U.S. EPA 1997) and predominantly below the current standard of 12 μg/m^3^ (U.S. EPA 2013).

For comparison with previous studies, we converted the percent change in mortality from our study to reflect a 10 μg/m^3^ increase. We found a 15.5% (95% CI: 0.8, 32.3%) increase in all natural-cause mortality for the entire population of New Jersey. By comparison, the HSC study reported an estimate of 13% (95% CI: 4, 23%), and its extended study reported a 14% (95% CI: 7, 22%) increase in mortality (Dockery et al. 1993; Lepeule et al. 2012). The ACS cohort, which examined the association among 500,000 residents of 51 cities found a 6% (95% CI: 2, 10%) increase in mortality (Pope et al. 1995, 2002). The NHS cohort, which examined the association with all-cause mortality among women, reported an increase of 26% (95% CI: 2, 54%) (Puett et al. 2009). Our results were at the higher end of the range compared with those of the cohort studies, possibly because we used a spatially resolved exposure model. The NHS study, which used geographically resolved exposure assessment, also tended to show a large effect size (Puett et al. 2009). Further, our model had a higher cross-validation *R* ^2^ than most land-use regression models. Hoek et al. (2008) summarized a number of land-use regressions. The highest *R* ^2^ of the model (typically higher than the cross-validation *R* ^2^) was 0.82 (Hoek et al. 2008). It is typical for most models to have an *R* ^2^ value < 0.7 (Hoek et al. 2008). The land-use regression used in the NHS study had cross-validation *R* ^2^ values of 0.77 and 0.69 for post- and pre-1999 periods, respectively (Yanosky et al. 2009). By comparison, our model had a cross-validation *R* ^2^ of 0.88, which produced exposure predictions with less measurement error. We found that the percent change in mortality among people > 65 years of age in New Jersey was 18.1% (95% CI: 0.6, 38.6%) for each 10 μg/m^3^ increase in long-term PM_2.5_. This estimate is larger than the estimated 4% (95% CI: 3, 6%) increase in all-cause mortality among Medicare beneficiaries residing in 4,568 ZIP codes (people ≥ 65 years old) during 2000–2005 (Zeger et al. 2008), which was calculated by using average PM_2.5_ concentrations measured by monitors within 6 mi of a ZIP code to approximate exposure. A lower exposure measurement error may be one of the reasons why our study found a larger effect of PM_2.5_. The sensitivity analysis (meta-analysis pooling within-census-tract effects) found a 3.7% (95% CI: 2.9, 4.5%) increase in mortality per IQR increase in PM_2.5_, suggesting that our result was close to the result obtained using the within-census-tract analysis.

We acknowledge that our study has limitations. First, we did not control for some of the differential changes over time across census tracts. Although temperature may be the strongest confounder between PM_2.5_ and mortality, the change over time in other variables such as the employment rate may also confound the relationship. Second, we did not measure individual-level predictors of mortality. Variations in these predictors within a census tract, however, cannot confound PM_2.5_ because they are not correlated with exposure (everyone in the tract has the same exposure in the same year). Nor can these variations confound associations between census tracts because there is no exposure contrast between tracts (because of the dummy variables for each tract). Furthermore, they cannot confound over time because the dummy variables for each year remove that pattern from outcome and exposure. For these variations to confound, their difference from the general trend by tract would have to be correlated with the differences around the trend in PM_2.5_, and we can see no mechanism that would produce this correlation. Although variations in the individual-level predictors cannot confound the association, we acknowledge that exposure misclassification can occur from assigning the same yearly averaged PM_2.5_ in census tracts for all residents. This variation in exposure for each individual around a small area should be Berksonian, which should not bias our estimates but would increase the confidence intervals. By comparison, cohort studies assigning exposure for each subject according to the date of death will not suffer from this problem if they have address-specific exposure. Moreover, our model is not susceptible to the typical ecological bias in which those who are exposed may not be those who develop the outcome; here, everyone within a census tract was assigned to the same geographically averaged exposure. Third, using PM_2.5_ at the census-tract level to assess exposure is still not as accurate as using PM_2.5_ predictions at the address level. Fourth, in our analysis, the strong control for spatial confounding and temporal trend using dummy variables for each census tract and each year substantially lowered the exposure contrast across tracts and over time, which potentially increased the standard error of effect of PM_2.5_. Fifth, the population in each census tract was likely to have changed from 2004 to 2009. Our analyses used population data from Census 2000 to approximate the population in 2004–2009, which may have reduced the accuracy of the estimates.

## Conclusions

Under the assumption that no variable changing differentially over time across census tracts other than seasonal temperatures could confound the association, we found causal associations between PM_2.5_ and all natural-cause mortality. The effect estimates of PM_2.5_ from our analyses were comparable to those of previous cohort studies, but on the higher end of the range. The association was modified by seasonal temperatures and by ecological SES variables.
